# Low-Complexity Repetitive Epitopes of *Plasmodium falciparum* Are Decoys for Humoural Immune Responses

**DOI:** 10.3389/fimmu.2020.00610

**Published:** 2020-04-15

**Authors:** Nan Hou, Ning Jiang, Yu Ma, Yang Zou, Xianyu Piao, Shuai Liu, Qijun Chen

**Affiliations:** ^1^NHC Key Laboratory of Systems Biology of Pathogens, Institute of Pathogen Biology, Chinese Academy of Medical Sciences & Peking Union Medical College, Beijing, China; ^2^Key Laboratory of Livestock Infectious Diseases in Northeast China, Ministry of Education, Key Laboratory of Zoonosis, College of Animal Science and Veterinary Medicine, Shenyang Agricultural University, Shenyang, China; ^3^The Research Unit for Pathogenic Mechanisms of Zoonotic Parasites, Chinese Academy of Medical Sciences, Shenyang, China; ^4^Beijing Key Laboratory for Research on Prevention and Treatment of Tropical Diseases, Beijing Tropical Medicine Research Institute, Beijing Friendship Hospital, Capital Medical University, Beijing, China

**Keywords:** *Plasmodium falciparum*, invasion, antigen, epitope, microarray, immune escape

## Abstract

Induction of humoural immunity is critical for clinical protection against malaria. More than 100 malaria vaccine candidates have been investigated at different developmental stages, but with limited protection. One of the roadblocks constrains the development of malaria vaccines is the poor immunogenicity of the antigens. The objective of this study was to map the linear B-cell epitopes of the *Plasmodium falciparum* erythrocyte invasion-associated antigens with a purpose of understanding humoural responses and protection. We conducted a large-scale screen using overlapping peptide microarrays of 37 proteins from the *P. falciparum* parasite, most of which are invasion-associated antigens which have been tested in clinical settings as vaccine candidates, with sera from individuals with various infection episodes. Analysis of the epitome of the antigens revealed that the most immunogenic epitopes were predominantly located in the low-complexity regions of the proteins containing repetitive and/or glutamate-rich motifs in different sequence contexts. However, *in vitro* assay showed the antibodies specific for these epitopes did not show invasion inhibitory effect. These discoveries indicated that the low-complexity regions of the parasite proteins might drive immune responses away from functional domains, which may be an instructive finding for the rational design of vaccine candidates.

## Introduction

Malaria elimination efforts have yielded outstanding achievements in the past 20 years, and malaria eradication by 2050 was proposed by the Lancet Commission (1). Although more than half of the world's countries are now malaria free (2), the currently available tools and approaches will not be sufficient to achieve the optimal goal of malaria eradication.

A potent vaccine has been believed to be the most cost-effective tool for reducing the negative impact of the disease on human health and is essential for complete malaria eradication. To date, more than 100 vaccine candidates have been investigated at different developmental stages. The world's first malaria vaccine, RTS,S/AS01, is now being deployed in a pilot roll-out in three African countries (3). However, the clinical protection obtained after immunization has not been as satisfactory as expected, although antigen-specific responses were sufficiently elicited (4, 5). Dispersion of immune responses by antigen diversity and functional compensation among family members of erythrocyte ligands have been speculated as reasons for the poor performance of the vaccine antigens. However, the immunogenic determinants of malarial antigens in connection with clinical protection are two critical aspects that have not been fully understood.

Invasion-associated antigens received wide attention as malaria vaccine candidates for invasion into erythrocytes is an essential step for the successful proliferation and transmission of plasmodial parasites (6, 7). In particular, merozoite antigens presented on the surface and that released upon contact of the merozoite with the erythrocyte are direct targets of naturally acquired humoural immunity and hence have been extensively investigated as potential candidates in vaccine development (8).

The objective of this study was to map the linear B-cell epitopes of the malaria antigens with a purpose of understanding humoural responses and protection. We conducted a large-scale linear epitope mapping using overlapped peptide microarrays to study the relationship between epitope characteristics and its antigenicity, and to find the clues resulting in high antigenicity but poor clinical protection. We screened the epitopes of 37 *Plasmodium falciparum* antigens, most of which are invasion-associated malaria vaccine candidates, with the sera of individuals of various infection histories. A clear epitope map of each antigen was generated. Epitopes containing repetitive sequences and glutamate-rich motifs were found highly antigenic and tended to be decoy epitopes that drive the host humoral immunity away from the functional domains.

## Materials and Methods

### Ethical Statement

All procedures performed on human samples were carried out in line with the tenets of the Declaration of Helsinki. Informed consent was obtained from every individual involved in this study, and all human samples were anonymized. All animal procedures in this study were conducted according to the animal husbandry guidelines of the Chinese Academy of Medical Sciences. The studies in both humans and animals were reviewed and approved by the Ethical Committee and the Experimental Animal Committee of the Chinese Academy of Medical Sciences, with Ethical Clearance Numbers IPB-2016-2 and CQJ16001.

### Sample Collection

A total of 289 patients suffering from falciparum malaria (FM) infection were recruited. All patients were experiencing fever (>37.5°C), blood samples were microscopically examined using Giemsa-stained thin blood smears and documented to be *P. falciparum* infection, then further confirmed by **nested** PCR (9). Among these patients, 60 were recruited in Libya from January to October 2012, 171 in Kachin state and Wa state, Burma from November 2006 to July 2011, and 58 in Beijing, Henan and Yunan, China, from September 2011 to January 2012. The sera samples of all patients were obtained before the patients received treatment. Sera samples from 144 healthy individuals (Healthy) were collected in Shenyang and Beijing, China, from September 2011 to January 2012 and were used as controls. More information about the individuals involved in this study is presented in Supplementary Table 1.

### Proteins and Peptides

**Figure 1:**
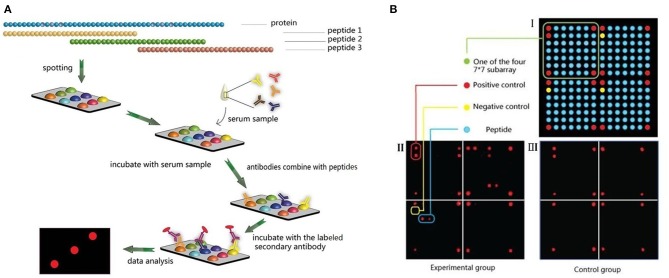
Microarray design and experimental procedures. **(A)** Peptides design and microarray detection procedure. **(B)** Spots arrangement on the microarrays.

**Figure 2:**
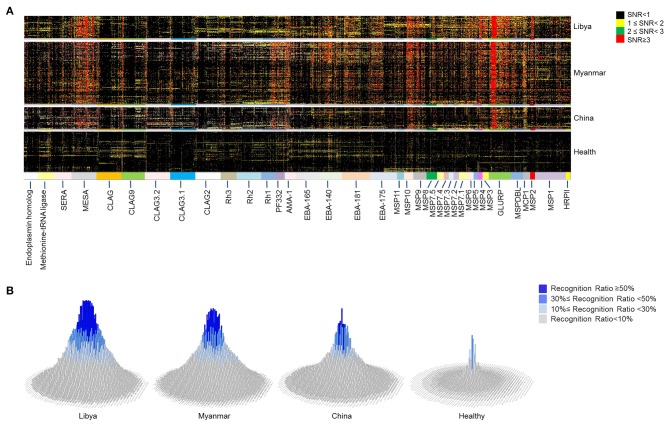
Microarray profile analysis for immunogenic epitopes of *Plasmodium falciparum* antigens. Microarray screening was carried out with 2,024 peptides mapping to 37 proteins of *P. falciparum* 3D7 strain. Sera from naturally exposed individuals from Libya (*n* = 60), Myanmar (*n* = 171), and China (*n* = 58) and healthy individuals from China (*n* = 144) were used to detect the antibody recognition of the peptides. **(A)** The heat map depicts the breadth and intensity of antibodies reactivity across sera samples. Each dot shows a signal to noise (SNR) value of one individual. The color of the dot indicates the intensity of the reaction with SNR. **(B)** The histograms show the prevalence rate of 2,024 peptides in different regions derived from the microarray profile. The height and color of the column indicate the prevalence rate of each peptide. Each column represents one peptide.

Thirty-seven *P. falciparum*-derived proteins were selected to explore the epitopes of the proteins, including apical membrane antigen (AMA)-1, cytoadherence linked asexual protein (CLAG) 3.1, 3.2, 8, and 9, erythrocyte binding antigen (EBA)-140, 165, 175, and 181, merozoite surface protein (MSP) 1, 2, 3, 4, 5, 6, 7.1, 7.2, 7.3, 7.4, 7.5, 8, 9, 10, and 11, merozoite surface protein duffy binding-like (MSPDBL), rhoptry-associated protein (Rh) 1, 2, and 3, merozoite capping protein 1 (MCP1), and endoplasmin, and a few mature parasite-derived antigens, such as *P. falciparum* 332 (PF332), histidine-rich protein (HRP) II, glutamate-rich protein (GLURP), mature-parasite-infected erythrocyte surface antigen [MESA, also called *P. falciparum* erythrocyte membrane protein 2 (PfEMP2)], serine repeat antigen (SERA) and methionine-tRNA ligase (7, 10). The amino acid sequence of each protein was derived from the protein database of NCBI (Supplementary File 1) and divided into consecutive peptides with a length of 30 amino acids, and each peptide had 15 amino acids overlapping with adjacent peptides (Figures 1A, 2A). In total, 2,053 peptides were generated, but only 2,024 peptides (Supplementary File 1) were successfully synthesized (GL Biochem, Shanghai, China). Thirteen extra synthetic peptides were designed and synthesized to verify the response to repetitive sequences.

### Microarray Construction

A microarray was prepared in a 100,000 grade clean room. Peptides were first dissolved to a concentration of 1 mg/ml with 30% acetonitrile solution (v/v, in Milli-Q water) as a stock solution. Then, the stock solution was diluted to 200 μg/ml with printing buffer [0.3 M phosphate-buffered saline (PBS), 0.2% glycerin, 0.01% Triton, and 1.5% mannitol] as the printing solution. SJ membranes (SJ Biomaterials, Suzhou, China) were used as supporting materials for their low background in serological assays, even without bovine serum albumin blocking (11). The membranes were first activated with the activation buffer (0.1 M 1-ethyl-3-(3-dimethylaminopropyl) carbodiimide and 0.1 M N-hydroxysuccinimide, both from Medpep, Shanghai, China) for 30 min and rinsed with Milli-Q water, then used for printing immediately. Microarrays were prepared using the non-contact printer sciFLEXARRAYER S1 (Scienion Co., Berlin, Germany) with one drop of 0.4 nL printing solution for each sample. All peptide samples were printed once to form a 7 × 7 × 4 array. For each subarray, the four corners of the square were positive controls spotted with Human IgG (DGCS-Bio, Beijing, China) at a concentration of 100 μg/ml. The first spot of the second line was a negative control with printing buffer, and the other 176 spots were all target samples (Figure 1B). All samples were printed in triplicate.

### Microarray Assays

Each serum sample was diluted with a dilution buffer (1% bovine serum albumin, 1% casein, 0.5% sucrose, 0.2% polyvinylpyrrolidone, and 0.5% Tween 20 in 0.01 M PBS, pH = 7.4). Then, 200 μl diluted serum was added onto each peptide microarray and incubated for 30 min on a shaker (150 rpm, 22°C). A microarray incubated with only the serum dilution buffer was included as a blank control. The microarray was then rinsed 3 times with TBST buffer (50 mM Tris, 150 mM NaCl, 0.05% Tween 20, pH 7.5) and incubated with 200 μl HRP-anti-human-IgG (1:5,000 diluted, ZSGB-Bio, Beijing, China) in Peroxidase Conjugate Stabilizer/Diluent (Thermo Fisher Scientific, Wilmington, USA) for another 30 min on the shaker (150 rpm, 22°C), followed by the same washing steps described above. Then, 15 μl SuperSignal ELISA Femto Maximum Sensitivity Substrate (Thermo Fisher Scientific) was added to the microarray to obtain chemiluminescence signals. The signal images were taken at a wavelength of 635 nm using an LAS 4000 imaging system (GE Healthcare, Uppsala, Sweden). The sera were diluted and tested for sensitivity and specificity at 1:100, 1:200, and 1:500 dilution, and 1:100 dilution was used in the eventual experiments due to its high sensitivity and low background (Supplementary Figure 1).

### Microarray Data Acquisition, Validation, and Analysis

The microarray data were extracted from the chemiluminescence emission with AMIA Toolbox (12). R_dot_ was the readout of the human IgG/peptide dot, and R_neg_ was the readout of the negative control dot. The Signal noise ratio (SNR) was defined by the following equation: (R_dot_ – R_neg_)/R_neg_. To validate the stability of the microarray, contrast experiments were carried out using the chips with the same peptides in the following groups: chips on two columns in one reactor, chips from two reactors, chips under the operation of two technicians, and chips from different lots (Supplementary Figure 2). The reaction intensity showed the concentration of the antibodies against the peptides in the sera. The intensity-cutoff values were determined at signal-to-noise ratio (SNR) = 2 to obtain a low level of false positives with high sensitivity. Thus, the reaction intensity was considered to be positive when the SNR ≥ 2.0. The prevalence rate (PR) was applied to depict the proportion of sera samples from a group that positively recognized a peptide and defined by the following equation: number of positive sera with SNR values ≥ 2/number of total sera from the appropriate group. Peptides with FM-PR ≥ 10%, Healthy-PR ≤ 10%, and FM-PR/Healthy-PR ≥ 2 (*p* < 0.05) were defined as positively recognized peptides. The peptides with FM-PR ≥ 50% and Healthy-PR <10% were considered to be highly antigenic peptides.

### Repetitive Sequence Analysis

Sequences that contained no less than two tandem repeats with more than three amino acids per repeat were considered to be repetitive sequences. Multiple sequence alignment was used to analyse the repetitive motifs of peptides. The alignments were conducted by using Clustalx 1.83 software (Genome Campus, Cambridgeshire, UK), and the alignments were edited by using Jalview (13).

### Invasion Inhibition Assays

The *P. falciparum* 3D7 strain was cultured and synchronized as previously described (14). Briefly, parasites were continuously cultured in malaria culture Media (MCM) in a candle jar at 37°C. The growth of the parasites was synchronized by treatment with 5% (w/v) D-sorbitol (Sigma, USA). Rabbit polyclonal antibodies were prepared at Beijing Protein Innovation (Beijing, China) by immunizing New Zealand white rabbits with Keyhole Limpet Hemocyanin (KHL)-coupled peptides (Supplementary Figure 3), which had the highest PR in the corresponding proteins or repetitive motifs. A total of 20 peptides were selected from 14 proteins that contain highly antigenic epitopes. Total IgG from sera of rabbits immunized with peptide were purified with the rProtein A Sepharose Fast Flow Kit (GE Healthcare, Uppsala, Sweden) according to the manufacturer's instructions. Total IgG from one rabbit was eventually used in each experiment with replicates. Complete culture medium (50 μl) and synchronized schizont-stage parasites (100 μl) were added to each well (0.5% parasitemia and 1% haematocrit) of 96-well U-bottom plates. Thereafter, 5 μl of test IgG was gently mixed into the indicated wells. The cultures were incubated at 37°C in a moist atmosphere of 94% N_2_, 1% O_2_, and 5% CO_2_. After an incubation of 40–42 h, the cells were harvested and transferred to tubes for the following steps and parasitemia was determined using flow cytometry. All samples were tested in duplicate.

### Measurement of Parasitemia

Thin smears of cultures were fixed in methanol and Giemsa stained for measurement of parasitemia by microscopy. The method used to detect parasitemia by flow cytometry was previously described (15). Briefly, 1–2 × 10^6^ red blood cells were fixed with 1 ml of PBS containing 0.025% (v/v) glutaraldehyde at room temperature for 20 min and permeated with 0.5 ml of PBS containing 0.01% saponin at room temperature for 5 min. Then, the cells were stained with propidium iodide (PI) at a final concentration of 10 μg/ml in PBS containing 2% FCS. The cells were detected and analyzed using a FACS Canto II flow cytometer (BD Biosciences, San Jose, CA, USA).

### Statistical Analysis

Data were analyzed using Excel 2010 and GraphPad Prism 5.0 (GraphPad, San Diego, CA). Two-tailed unpaired Student's *t*-tests were used to evaluate the immunoreactivity of the recognized peptides. Pearson and Spearman correlation analysis was used to calculate correlation coefficients. Values of *p* < 0.05 were considered to constitute significant differences.

## Results

### The Antigenic Epitopes of Critical *P. falciparum* Antigens Depicted by Peptide Microarray Screening

The proteins (Table 1) involved in parasite invasion of red blood cells were selected, and the sequences of these proteins were divided into consecutive peptides to explore the immunogenic epitopes. Each peptide has 30 amino acids, and 15 amino acids overlapped with adjacent peptides. The antigenicity of the epitopes was analyzed by microarray. Microarray screening of the peptide profile was carried out with 289 sera samples from patients suffering from FM and 144 healthy individuals as controls. Sixty FM patients were from Libya, while 171 were from Burma, and 58 were from China. The peptides were recognized with various reaction intensities, but most of them were poorly recognized by the sera (Figure 2A). Among the 2,024 peptides of the microarray profile, only 39.2% (794 peptides) were positively recognized (Supplementary File 2). The peptides that showed high reaction intensity tended to have a high prevalence of specific antibodies (Figure 2 and Supplementary Figure 4).

**Table 1:** The invasion-associated antigens of *Plasmodium falciparum* blood stage parasites involved in microarray screening.

**Stage-association**	**Protein**	**NCBI protein ID**	**Description**
Merozoite-associated proteins	Apical membrane antigen (AMA)-1	XP_001348015.1	An important vaccine candidate that is expressed in mature stage parasites and is essential for invasion (16–19)
	CLAG	XP_002808744.1	A strictly conserved family which play roles in merozoite invasion and infected cell adherence (20)
	CLAG3.1	XP_001351100.1	
	CLAG3.2	XP_001351099.1	
	CLAG9	XP_001352222.1	
	CLAG2	XP_001349709.1	
	Erythrocyte binding antigen (EBA) 140	XP_001349859.1	Members of erythrocyte binding-like family (EBL) of proteins involved in tight junction formation during invasion of red blood cells and as potential vaccine candidate for malaria (21–25)
	EBA165	XP_001351546.1	
	EBA175	XP_001349207.2	
	EBA181	XP_001350957.1	
	Endoplasmin homolog	XP_001350620.1	A protein with heat shock protein (Hsp) 90 domain which may serve as a molecular clamp in the binding of ligand proteins to Hsp90 (26)
	Merozoite capping protein 1 (MCP1)	XP_001347552.1	A 60-kDa protein participating in merozoite invasion of erythrocytes by facilitating attachment or movement of the junction along the parasite cytoskeletal network (26)
	Merozoite surface protein (MSP)1	XP_001352170.1	GPI-anchored proteins expressed on the merozoite surface, most of which are essential for parasite survival (10, 27). MSP-1 is the most abundant protein of GPI-anchored proteins. Vaccines with MSP-1, 2 and 3 are now being tested in clinical Phase trials (28–32)
	MSP2	XP_001349578.1	
	MSP3	XP_001347629.1	
	MSP4	XP_001349580.1	
	MSP5	XP_001349579.1	
	MSP6	XP_001347630.1	
	MSP7.1	XP_001350074.1	
	MSP7.2	XP_001350075.1	
	MSP7.3	XP_002809050.1	
	MSP7.4	XP_002809050.1	
	MSP7.5	XP_001350080.1	
	MSP8	XP_001351583.1	
	MSP9	XP_001350683.1	
	MSP10	XP_966190.1	
	MSP11	XP_001347636.1	
	Merozoite surface protein duffy binding-like (MSPDBL)	XP_001347632.1	A protein with Duffy binding-like (DBL) domain Localized on the merozoite surface and binding of merozoites with erythrocytes during invasion (33)
	Rhoptry-associated protein (RAP)1	XP_001348275.1	Rhoptry bulb proteins localsing to the parasite-host cell interface and rhoptry-associated protein complex facilitates the survival of the parasites (34)
	RAP2	XP_002808967.1	
	RAP3	XP_001351928.1	
Mature parasite-derived proteins	Glutamate-rich protein (GLURP) or	XP_001347628.1	The antigen of a blood-stage vaccine of malaria, which is an exoantigen expressed at all stages of development in the parasite life cycle in human host (35–38). Phase Ib trial of the vaccine candidate GMZ2 with Glurp and MSP has been finished (39)
	Histidine-rich protein (HRP) II	XP_002808743.1	A valuable protein for diagnosis of malaria since it is produced by ring and trophozoite-stage parasites and secreted into plasma (40, 41)
	Mature-parasite-infected erythrocyte surface antigen (MESA)	XP_001351567.1	A highly repetitive protein plays a major role in cytoadherence of infected erythrocytes by binding with erythrocyte membrane skeletal protein 4.1 (42–44)
	Methionine-tRNA ligase	XP_001347624.1	A protein with Glutathione-S-transferase (GST)-like domains which are involved in protein-protein interaction. The protein localizes to apicoplasts in asexual stages of parasites and play important roles on parasite growth (45, 46)
	*P. falciparum* 332 (PF332)	XP_001348162.2	A Large protein locating on the surface of mature schizonts plays critical roles in both invasion and sequestration with the Duffy-binding-like domain (47, 48)
	Serine repeat antigen (SERA)5	XP_001349586.1	An abundant late-trophozoite and schizont stage antigen with limited polymorphism which is being tested in clinical Phase trials (49, 50)

Furthermore, the reaction intensity (Figure 2A) and prevalence (Figure 2B) of the antibodies to the specific epitopes varied with the intensity of malaria transmission. For most of the peptides, the antibody reaction intensity and prevalence gradually decreased from high-endemicity areas to low-endemicity areas. These findings are consistent with previous studies (51). However, a large number of peptides had higher antibody reaction intensity and prevalence in Burma, the relatively low-endemic area, than in Libya. These peptides were mainly attributed to AMA-1, CLAG family members, EBA family members, MSP family members, PF332, Rh1, Rh2, and SERA5 (Supplementary File 3). Thus, the humoural response is not always related to the transmission intensity.

**Figure 3:**
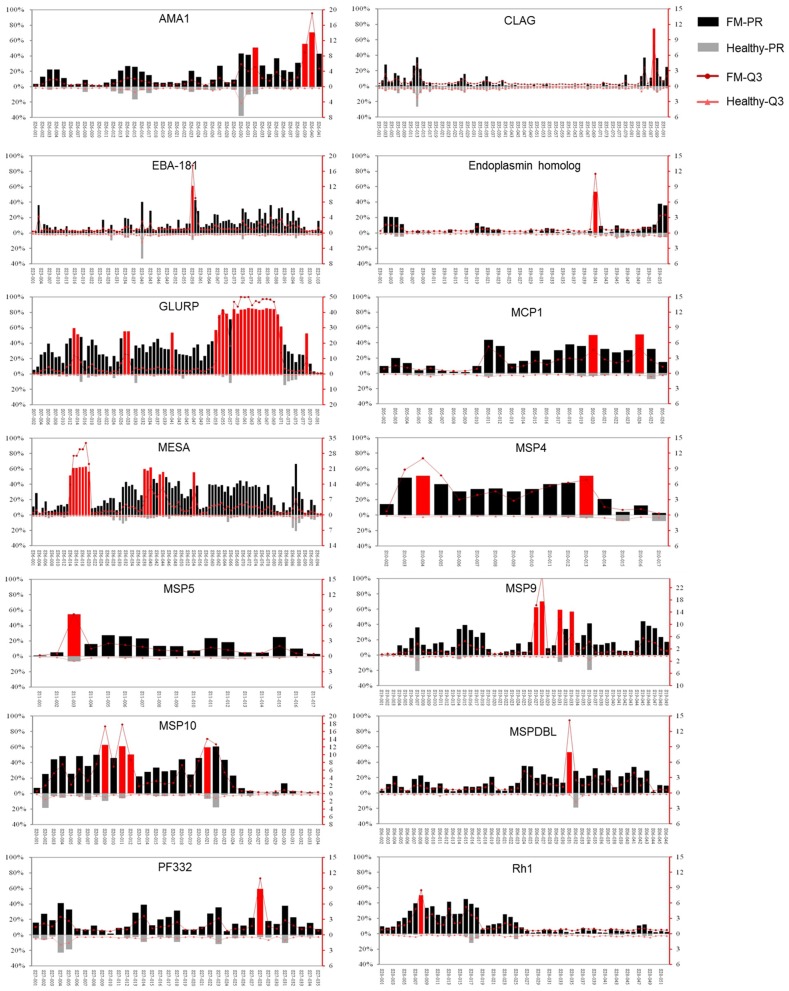
Immunoreactivity profiles of the 14 proteins that contained highly antigenic peptides. The reaction intensity and prevalence rate (PR) of the specific antibodies against peptides from the antigens were detected by microarray with sera from falciparum patients (FM) and healthy individuals (Healthy). The PR for the appropriate group (left Y axis) and the reaction intensity with the third quartile of signal noise ratio (Q3, right Y axis) were showed. Each pair of columns or dots represents one peptide. Peptides with prevalence rates ≥50% in FM patients, <10% in healthy individuals and FM-PR/Healthy-PR ≥ 2 (*p* < 0.05) were highlighted in red. The upper columns or dots represent FM patients (FM-PR or FM-Q3), and the below represent healthy individuals (Healthy-PR or Healthy-Q3). The profiles of proteins were represented in the name order.

Among the 37 proteins, only 14 proteins, GLURP, MESA, AMA-1, CLAG, EBA-181, endoplasmin homolog, MCP1, MSP4, 5, 9, and 10, MSPDBL, PF332, and Rh1, contained peptides with FM-PR ≥ 50%, Healthy-PR <10% and FM-PR/Healthy-PR ≥ 2 (*p* < 0.05, Figure 3). These peptides with high PR tended to have high reaction intensity with the third quartile of SNR (Q3); thus, they were considered to contain highly antigenic epitopes. Among the 14 proteins, only GLURP and PfEMP2/MESA contained highly antigenic epitopes with a relatively wider distribution in the molecules, whereas the reactivity of the other proteins was restricted to only one or a few peptides (Figure 3).

### Repetitive Sequences With Distinct Amino Acid Contexts Were Predominantly Immunogenic

**Figure 4:**
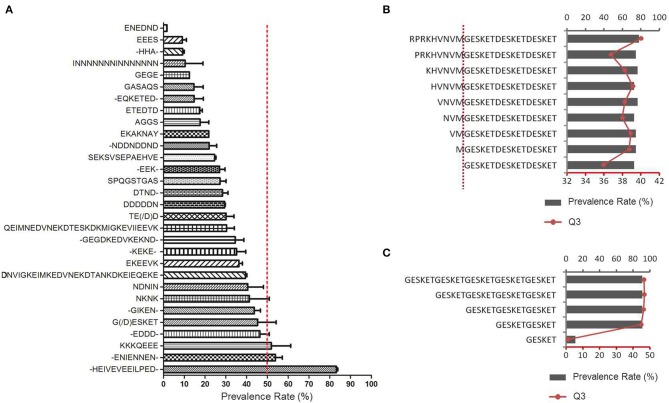
Antigenicity of repetitive peptides. **(A)** The prevalence rates of antibodies recognizing different peptides with the repetitive motifs were derived from the microarray profile. The 30 types of repetitive sequences were represented in rows in ascending order of the average PR of all the peptides containing the motif (Mean ± SD). - indicates varied amino acid. The red dotted line indicates the 50% prevalence rate. **(B,C)** Thirteen extra synthetic peptides were printed onto the microarray chips and detected by sera from African FM patients. **(B)** Influence of the adjacent amino acids on the immunoreactivity of peptides with the G(D)ESKET motif **(C)** The number of G(D)ESKET required for eliciting optimal humoral responses. The prevalence rate for appropriate group (columns, up X axis) and the reaction intensity with the third quartile of signal noise ratio (Q3, red dot, low X axis) were showed.

The sequences of positively reactive peptides were analyzed to reveal the characteristics of the immunogenic determinants of the antigens. Surprisingly, most of the immunodeterminants were located in the low-complexity regions composed of distinct repetitive amino acid motifs (Table 2). The proportion of repetitive peptides rose perpendicularly with the increase in PR, and 11 peptides (91.7%) were repetitive peptides among the 12 peptides with a PR ≥ 80%. Although most of the highly antigenic peptides were repetitive peptides, not all of the repetitive sequences were highly antigenic. Among the 2,024 peptides of the 37 proteins, 139 peptides from 17 proteins contained repetitive motifs (Figure 4A and Supplementary File 4). Of those repetitive motifs, 6 repetitive motifs, -HEIVEVEEILPED-, -ENIENNEN-, KKKQEEE, -EDDD, G(/D)ESKET, and NKNK were located in peptides with high antigenicity (Figure 4A). Different peptides with the tandem repeat motif -HEIVEVEEILPED- were most immunogenic, with a PR value of 83.2 ± 2.5%, while the peptides with repeating motif ENEDND had a PR value of only 1.7 ± 0.0% for sera samples from malaria-exposed individuals.

**Table 2:** Characteristics of positively recognized peptides.

**Prevalence rate (PR)**	**No. peptides**	**Repetitive peptides**		**E-rich peptides**		**E-rich repetitive peptides**	
		**No**.	**Proportion of total peptides (%)**	**No**.	**Proportion of total peptides (%)**	**No**.	**Proportion of repetitive peptides (%)**
≥80%	12	11	91.7	10	83.3	10	90.9
<80%, ≥60%	16	9	56.3	8	50.0	7	77.8
<60%, ≥50%	29	10	34.5	5	17.2	3	30.0
<50%, ≥40%	46	15	32.6	7	15.2	5	33.3
<40%, ≥30%	116	27	23.3	17	14.7	12	44.4
<30%, ≥20%	211	25	11.8	15	7.1	7	28.0
<20%, ≥10%	364	21	5.8	19	5.2	9	42.9
Total	794	118	1.5	81	1.0	53	44.9

*Peptides with FM-PR ≥ 10%, Healthy-PR <10%, and FM-PR/Healthy-PR ≥ 2 were considered to be positively recognized peptides. Glutamic (E)-rich cutoff = 30%*.

To determine the significance of the amino acids within or surrounding the motif in their effect on the antigenicity, different peptides were constructed containing at least one GESKET motif and a decreased number of non-repetitive amino acids according to the peptide sequence of the MESA protein, RPRKH VNVMG ESKET GESKE TGESK ETGES, or with decreased tandem repeats of GESKET, and then screened with the same set of patient sera. The addition of any amino acids to the core repetitive sequence did not obviously alter the antibody recognition rate or reaction intensity (Figure 4B), and additional units beyond two repeats did not add any power for recognition (Figure 4C). Thus, a sequence containing two tandem repeats of the motif will be sufficient to form a B cell epitope and can elicit strong humoural immune responses.

Another feature of the highly immunogenic polypeptides was the enrichment of glutamic acid. Among the 2,024 peptides, 94 were glutamate-rich, in which glutamic acid accounted for over 30% of the amino acid composition, and 23 (24.5%) glutamate-rich peptides were recognized by over 50% of sera samples from malaria-exposed individuals (Supplementary File 5). Furthermore, the proportion of glutamate-rich peptides increased to 83.3% when the PR was over 80% (Table 2). Among the 11 repetitive polypeptides with a PR over 80% in sera samples from the FM patients, 10 (90.9%) were glutamate-rich (Table 2). For all 2,024 peptides, the content of glutamic acid was positively correlated with the PR in sera samples from malaria-exposed individuals (Pearson *r* = 0.1090, *p* < 0.0001, Supplementary Figure 5).

### The Antibodies Specific for the Highly Antigenic Epitopes Failed to Interfere With Parasite Invasion

**Figure 5:**
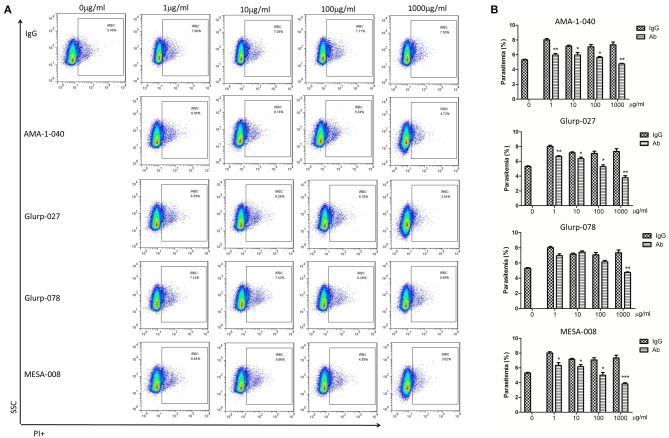
Invasion inhibition assays with total IgG containing peptide-specific antibodies. Highly synchronized schizont-stage parasites of PF3D7 strain were cultured in the presence of total IgG containing polyclonal antibodies (Ab) and IgG control derived from naïve rabbits. Parasitemia was determined using flow cytometry after 40–42 h of cultivation. **(A)** Representative dot plots showing the frequency of PI+ infected red blood cells (iRBC). **(B)** Histograms comparing the parasitemia between antibody-treated group and IgG control group. The results are representative of 3 independent experiments, with data indicating the mean + SD. *p < 0.05, **p < 0.01, and ***p < 0.0001. *Comparison to the corresponding naïve IgG group.

To determine whether the epitope-specific antibodies are protective, invasion inhibition assays were undertaken to investigate the neutralization effect of the total IgG from peptide-immunized rabbits. In total, 20 peptides from 14 proteins that contain highly antigenic epitopes were selected as immunogens for generation of specific antibodies. Peptide-specific polyclonal antibodies were generated, and total IgG was purified. Antibodies specific to the peptide (MESA-008) from the red blood cell-binding sites of MESA with low antigenicity were also applied to compare the neutralization activity of the antibodies specific for the functional domain with that of the antibodies specific for immunodeterminants. Normal rabbit IgG was used as a negative control. Only antibodies specific for AMA-1-040, GLURP-027, GLURP-078, and MESA-008 showed invasion inhibitory effect (Figure 5), and the three antibodies specific for AMA-1-040, GLURP-027, and GLURP-078 showed a weaker effect than the antibodies specific for MESA-008. Most of the antibodies specific for the highly antigenic peptides showed no inhibitory effect on parasite invasion (Supplementary Figure 6).

## Discussion

Invasion into erythrocytes by extracellular merozoites is an essential step in the development and proliferation of malarial parasites (6, 7). In recent decades, various antigens have been explored for the development of blood-stage vaccines. However, progress has been limited, which has been attributed to antigen polymorphisms, antigenic variation and functional complementation (7, 10). In previous studies, the criteria for vaccine candidate selection have been mainly based on the immunogenicity of antigens in eliciting high antibody titres, and CSP-based vaccines are the best example (52, 53). Lessons have indicated that apart from the importance of cellular immune responses, antibodies that can neutralize the infectivity of the parasite are critical. In this study, we used overlapping peptide microarrays to investigate the immunogenic epitopes of 37 *P. falciparum* antigens, most of which are essential proteins in parasite invasion and have been extensively explored in malaria vaccine development.

To date, epitopes analyses have either been based on limited structural data or sequence data alone. The structural studies located the position of epitopes within the protein conformation (54). However, studies on discontinuous B-cell epitopes are laborious, time consuming and provide limited information. The overlapping peptide array in our study, with 2,024 peptides derived from 37 malaria antigens, presented abundant information in one study and present a fine map of linear B-cell epitopes that elicit humoral immune responses.

Here, surprisingly, of the highly immunogenic regions of the well-recognized antigens, most of the epitopes were located in the low-complexity regions composed of repetitive amino acid motifs (Table 2). The proportion of repetitive peptides sharply increased and reached 91.7% when the antibody PR was over 80% (Table 2). Tandemly repeated amino acid sequences are characteristic of many malaria parasite proteins, and a multitude of other higher eukaryotic parasites, such as *Toxoplasma, Leishmania, Trypanosoma*, and *Schistosoma*, share this common feature, and it has been proposed that antigens with sequence repeats are dominant natural immunogens (55).

Enrichment of glutamic acid was another feature of the highly immunogenic polypeptides. Most peptides with a high antibody reaction intensity and PR were glutamate-rich sequences, and the proportion of glutamate-rich peptides sharply increased and reached 83.3% when the PR was over 80% (Table 2). Furthermore, the repetitive and glutamate-rich characteristics were strongly correlated with the immunogenicity of the peptides (Table 2 and Supplementary Figures 4, 5). Some glutamate-rich proteins of *Plasmodium* were found to be antigenic and had a serological cross-reaction. GLURP, the glutamate-rich protein of *P. falciparum*, is expressed in both liver and blood stage parasites (35). Previous studies have found a strong correlation between the levels of GLURP-specific IgGs and protection against malaria attacks (36, 37). The human immune response is primarily directed to R2 (aa 705-1178), where the highly immunogenic repeats are located, and this region of GLURP has been developed primarily as a blood-stage vaccine (38). In addition to *Plasmodium*, many other pathogens, such as *Babesia gibsoni* (56), *Entamoeba histolytica* (57), *Pneumocystis carinii*, and *Mycobacterium tuberculosis* (58), also have immunogenic glutamate-rich proteins, and some of them were evaluated as new diagnostic tools or vaccine candidates. Additionally, many allergens from plants (59–61) and some self-antigens that lead to autoimmune disease (62, 63) were also glutamate-rich antigens. Thus, it is likely that there are many glutamate-rich proteins that are highly immunogenic in various pathogen species, especially in parasites.

The function of conserved, tandemly repeated regions within proteins has been discussed for decades and is still not clear (64, 65). Proteins with repetitive structures have already been used for vaccines. These proteins, including the circumsporozoite protein (CSP), were initially spotted by immune-screening of cDNA libraries and later included in vaccine development (52, 53). However, except for the most advanced pre-erythrocytic vaccine candidate RTS,S, the progress of vaccines based on these antigens has not been very satisfactory. Hypotheses such as antigenic variation and functional complementation with alternative proteins that allow the parasite to evade the protective immune responses of the host have been proposed (5, 66), but could not explain the failures of vaccines targeting the early-stage parasite.

A direct invasion inhibitory effect of antibodies on the functional domains of malaria antigens has been observed in many studies. Anti-AMA1 polyclonal and monoclonal antibodies block parasite invasion *in vitro* (16–18). Monoclonal antibodies against the epitope mapped to the receptor binding sites of EBA-175 could block *P. falciparum* erythrocyte invasion (25). Our results also found that antibodies against the erythrocyte binding site of PfEMP2/MESA could inhibit parasite invasion. MESA has been recognized as a membrane-anchored protein that is expressed in the trophozoite stage of the parasite and contains 7 distinct repeat regions that cover over 60% of the protein (42, 43). The distribution of the repeats in the molecule is highly conserved [Figure 4B and Supplementary Figure 7; (67)]. The binding domain of MESA with the erythrocytic protein 4.1 was mapped to a 19-residue region near the N-terminus of the protein (44).

However, antibodies against the high-antigenicity and low-complexity epitopes represented by the microarray seemed to have no neutralizing effect. Since the sera used in this study were derived from patients, particularly the Chinese patients returning from African regions who were suffering from *P. falciparum* infection with at least one malaria episode, the antibodies specific for the repetitive antigenic motifs were obviously not protective. The *in vitro* assay revealed that most antibodies to the highly antigenic epitopes showed no inhibitory effect on parasite invasion (Supplementary Figure 6). The antibodies of AMA-1-040, GLURP-027, and GLURP-078, which displayed slight invasion inhibition effect, were all located outside the low-complexity region of the protein sequences (Figure 5). When trying to build three-dimensional structures of these proteins with SWISS-MODEL [https://swissmodel.expasy.org/interactive; (68)] to predict the location of the highly antigenic epitopes, however, these epitopes were all located in the regions without homolog models, indicating that these regions are likely within the non-functional domains. Thus, it is hypothesized that antibodies to the low-complexity regions might not be able to interfere with the functionality of the host molecules. Furthermore, these highly antigenic peptides had very low polymorphism in different isolates of *P. falciparum* (Supplementary Figure 7), indicating these sequences may serve as decoy epitopes to attract host humoural immunity.

Previous studies reported that antibodies of highly antigenic epitopes could block parasite invasion through an antibody-dependent cell inhibition (ADCI) mechanism (69, 70). However, most of these studies were carried out *in vitro* with monocytes from healthy individuals. Our study with *P. berghei* ANKA (PbANKA)-infected C57BL/6 mice revealed that although monocytes/macrophages facilitated parasite clearance in the early stage of infection, their activity and quantity declined soon post-infection (71). Some parasite antigens were even reported to suppress monocyte/macrophage activation (72). Furthermore, phagocytic receptors of non-opsonic phagocytosis, such as CD36 and ICAM-1, were sharply decreased post-infection or even deficient in malaria-endemic regions (73–75). Thus, even though these antibodies could interfere with parasite invasion through ADCI *in vitro*, their effect *in vivo* remains uncertain, considering the very short extracellular period of time before parasite invasion.

Collectively, we found that *P. falciparum* antigens that contain repetitive sequences and glutamate-rich motifs are highly antigenic. The motifs with a confined sequence context drove humoural responses to the protein regions that are likely not functional. Thus, malaria parasites, especially *P. falciparum*, may have developed a mechanism of immune evasion with the decoy epitopes that drive the host humoural immunity away from the recognition of the functional domains of these antigens (64, 65). These findings highlight the importance of determination of antigens that can elicit protective immune responses.

## Data Availability Statement

All datasets generated for this study are included in the article/Supplementary Material.

## Ethics Statement

All procedures performed on human samples were carried out in line with the tenets of the Declaration of Helsinki. Informed consent was obtained from every individual involved in this study, and all human samples were anonymized. All animal procedures in this study were conducted according to the animal husbandry guidelines of the Chinese Academy of Medical Sciences. The studies in both humans and animals were reviewed and approved by the Ethical Committee and the Experimental Animal Committee of the Chinese Academy of Medical Sciences, with Ethical Clearance Numbers IPB-2016-2 and CQJ16001. The patients/participants provided their written informed consent to participate in this study.

## Author Contributions

QC conceived and designed experiments. NH, NJ, and YM performed the majority of the experiments. NH and QC analyzed the data and wrote the manuscript. YZ, XP, and SL performed some experiments.

### Conflict of Interest

The authors declare that the research was conducted in the absence of any commercial or financial relationships that could be construed as a potential conflict of interest.
