# Supplementary material for: Low-Complexity Repetitive Epitopes of Plasmodium falciparum Are Decoys for Humoural Immune Responses
Source: Front Immunol. 2020 Apr 15;11:610. doi: 10.3389/fimmu.2020.00610 (PMC7174639; doi:10.3389/fimmu.2020.00610)
Supplement: Supplementary file 1 [file Data_Sheet_1.docx]

Supplementary Figures


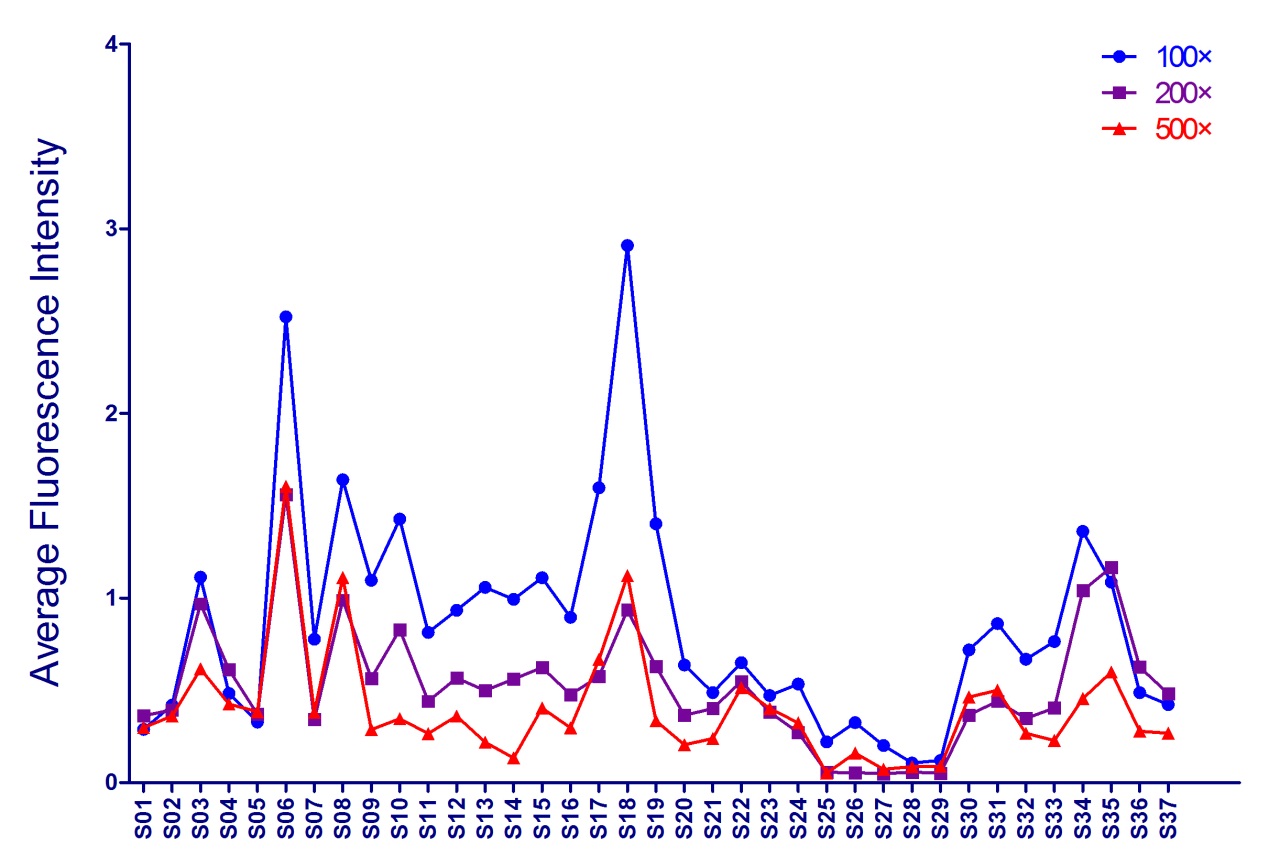


Supplementary Figure 1. The dilution ladder of sera in microarray detection.

71 sera samples (60 African natives and 11 China migrants from Africa) were used for pre-experiment to confirm the most proper serum concentration with relatively high sensitivity and low background. The sera were diluted with serum dilution buffer at 1:100, 1:200 and 1:500. Chips with 2024 peptides were detected and the signals were analyzed.


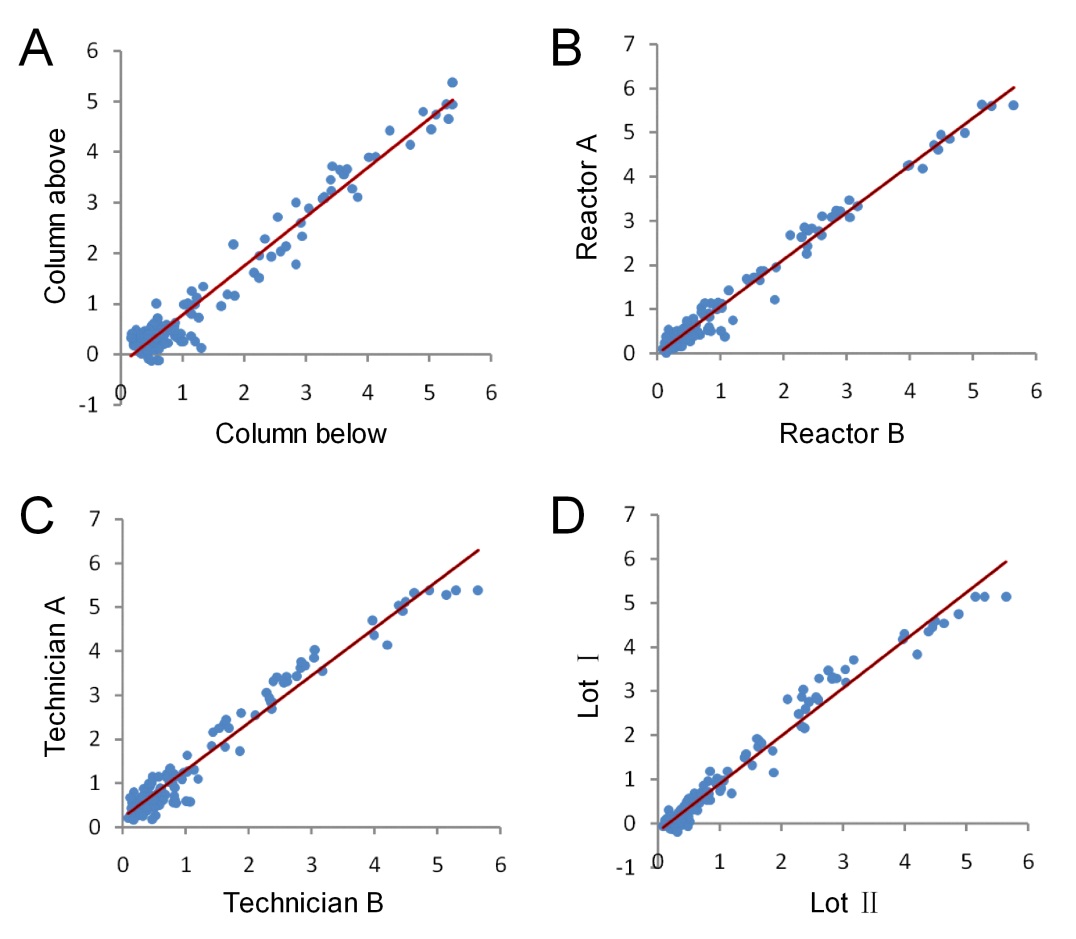


Supplementary Figure 2. Microarray validation assays.

To validate the stability of the microarray, contrast experiments were carried out using the chips with same peptides in following groups: (A) chips on two columns in one reactor, (B) chips from two reactors, (C) chips under operation of two technicians, and (D) chips from two Lots.


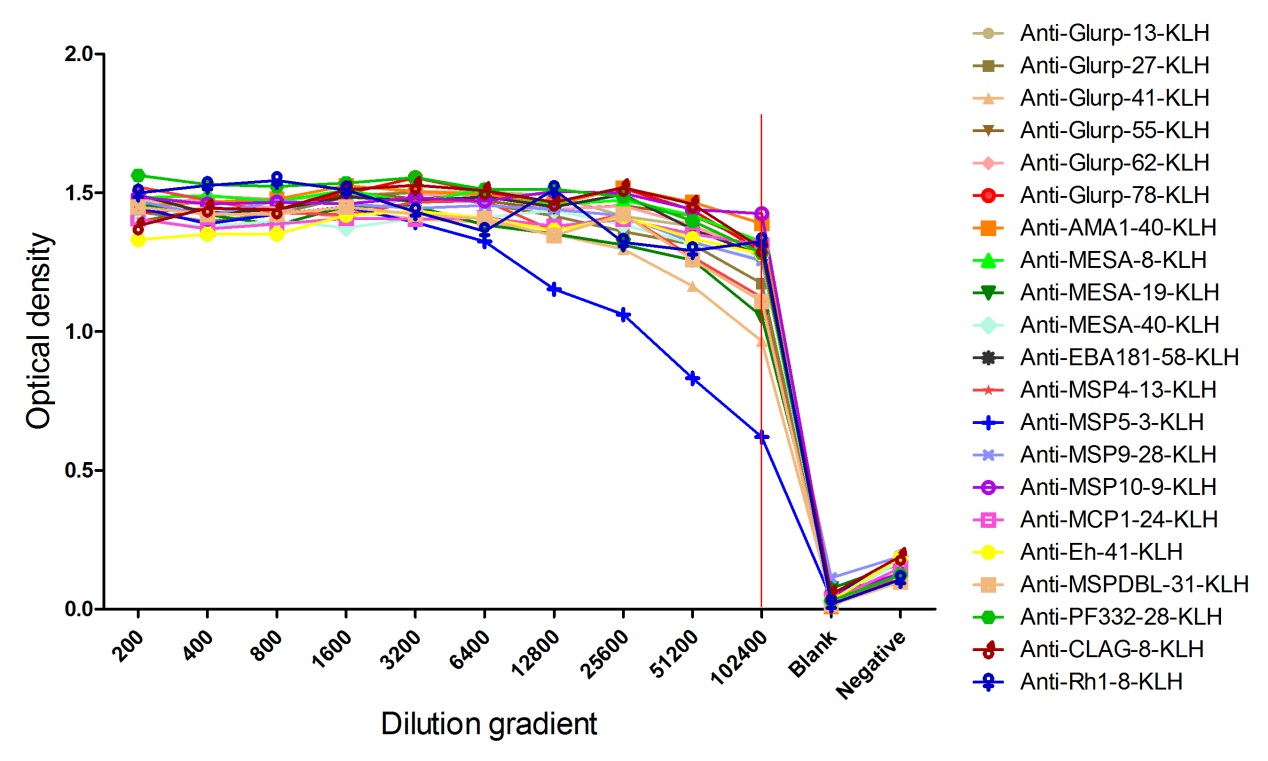


Supplementary Figure 3. The titer of rabbit antibodies.

New Zealand white rabbits were immunized with KHL-coupled peptides. The titers of antibodies in rabbit serum were detected by ELISA with BSA-coupled peptides after the third booster immunization. Double wavelength (450nm and 630nm) readout was performed to detect the absorbance on a microplate reader. Antibiotic titer was defined as the dilution which indicated to half of the highest optical density.


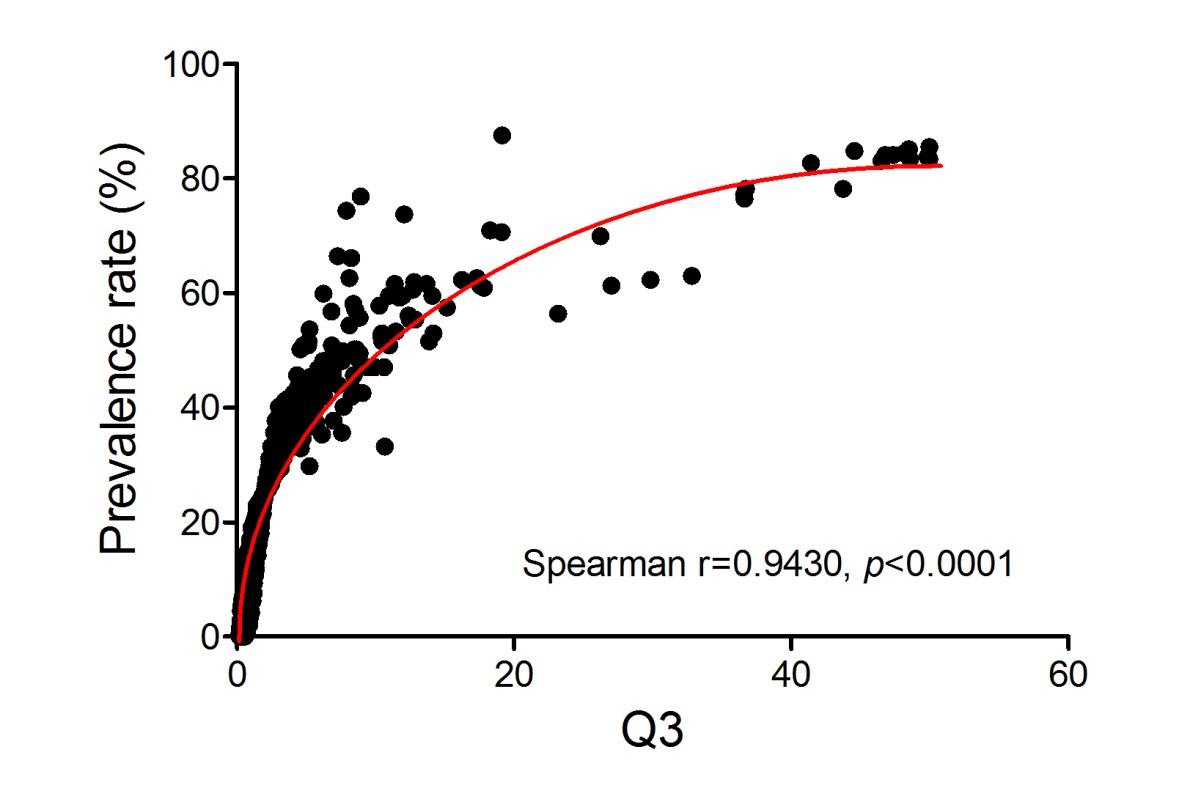


Supplementary Figure 4. The correlation analysis of the third quartile of signal noise ratio (Q3) of antibody intensity with the prevalence rate (PR) of the peptides derived from the microarray.

The reaction intensity and prevalence rate of the specific antibodies against peptides from the antigens were detected by microarray with sera from falciparum patients (FM). The relationship of PR and the reaction intensity with Q3 were evaluated by Spearman correlation analysis. Each dot represents one peptide.


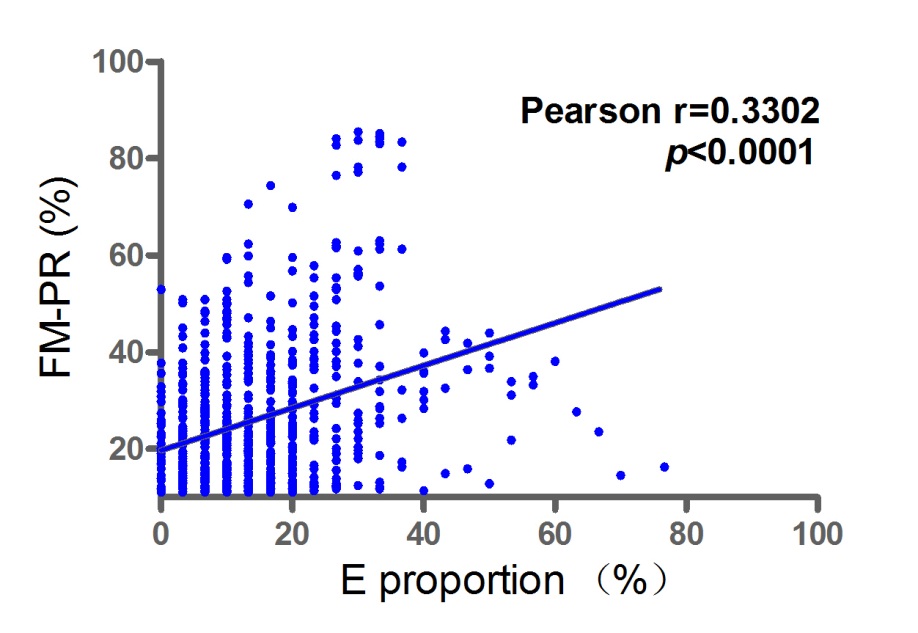


Supplementary Figure 5. The influence of glutamine (E) content of the peptides on the antibody prevalence rate (PR).

The reaction intensity and prevalence rate of the specific antibodies against peptides from the antigens were detected by microarray with sera from falciparum patients (FM). The relationship of PR and the proportion of glutamine in the peptide amino acid content were evaluated by Pearson correlation analysis. Each dot represents one peptide.


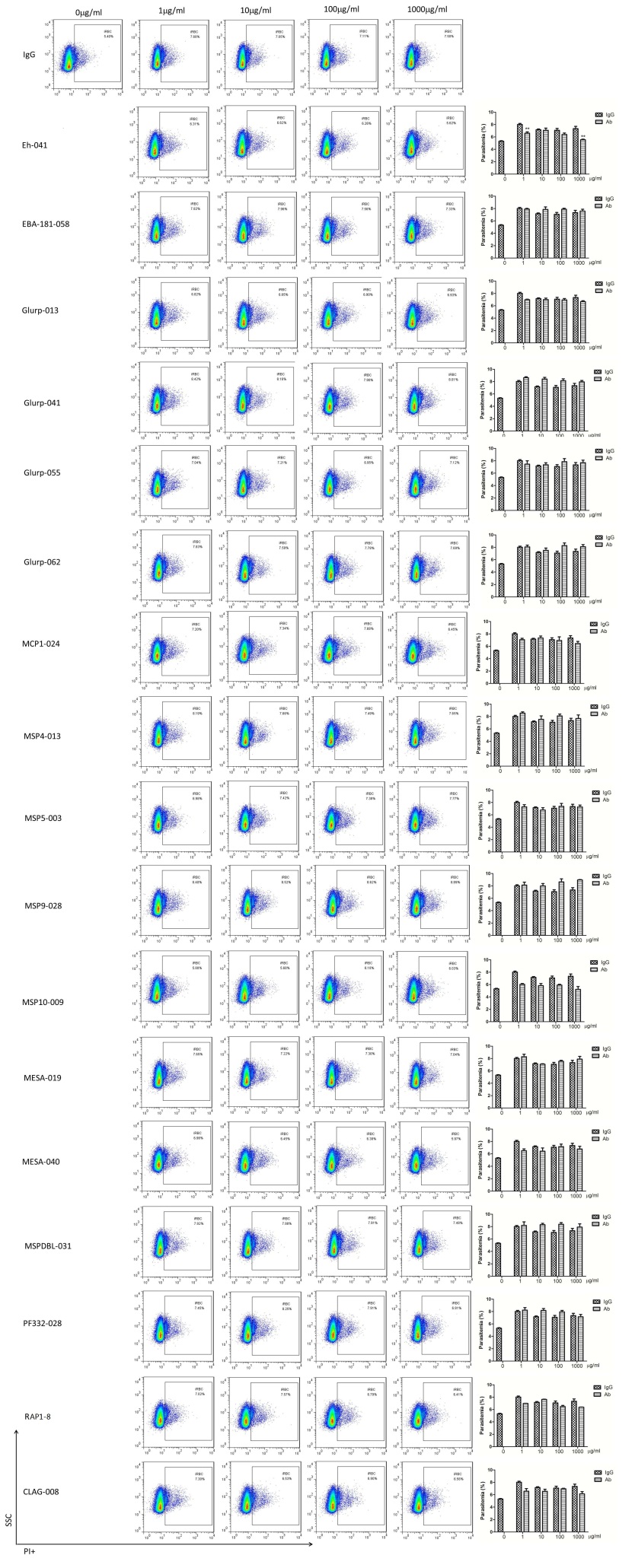


Supplementary Figure 6. Peptide-specific antibodies did not inhibit parasite invasion.

Highly synchronized schizont-stage parasites of PF3D7 strain were cultured in the presence of purified total IgG containing polyclonal peptide-specific antibodies (Ab) or IgG control. Parasitemia was determined using flow cytometry after 40-42 hours of cultivation. Representative dot plots (Left panels) showed the frequency of PI+ infected red blood cells (iRBC). Histograms (right panels) compared the parasitemia between antibody-treated group and IgG control group. The results are representative of 3 independent experiments, with data indicating the mean + SD. **, p<0.01. ** indicates comparisons to IgG 0 μg/ml group.


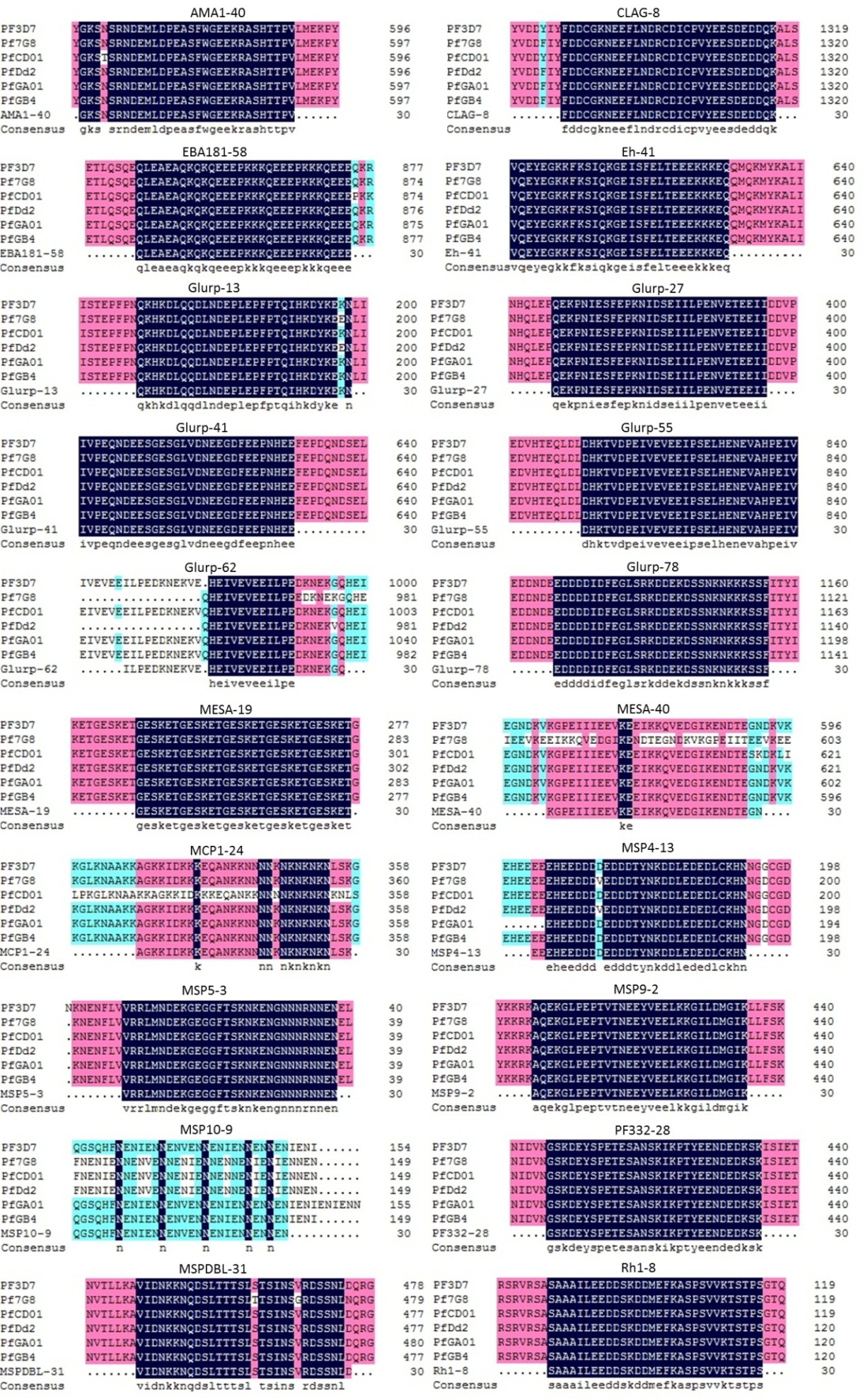


Supplementary Figure 7. Low polymorphism of the highly antigenic peptides. The 20 highly antigenic peptides chosen for invasion inhibitory assay were belonged to 14 proteins. The amino acid sequence of the proteins in 6 different isolates were derived from PlasmoDB (<https://plasmodb.org/plasmo/>), alignments were done with Cluster 3.0 to show the polymorphism of these peptides.
