# Supplementary material for: Low-Complexity Repetitive Epitopes of Plasmodium falciparum Are Decoys for Humoural Immune Responses
Source: Front Immunol. 2020 Apr 15;11:610. doi: 10.3389/fimmu.2020.00610 (PMC7174639; doi:10.3389/fimmu.2020.00610)
Supplement: Supplementary file 2 [file Data_Sheet_2.docx]

**Supplementary Table 1** Participant information

| **Subjects** | **FM** | | | | | **Healthy** |
| --- | --- | --- | --- | --- | --- | --- |
| **Input location** | Libya | Africa | China | Burma | | China |
| **Native location** | Libya | China | China | China | Burma | China |
| **Age mean^*^ (years)** | 26.8±8.5 | 33.5±7.9 | 34.5±9.1 | 30.0±9.0 | 25.3±9.7 | 29.7±7.6 |
| **Age range (years)** | 12-45 | 24-51 | 19-55 | 16-49 | 10-58 | 15-58 |
| **Male/Female** | 36/24 | 11/0 | 44/3 | 27/0 | 111/33 | 113/31 |
| **First infection/Reinfection** | 5/55 | 11/0 | 38/9 | 27/0 | 18/126 | 0/0 |
| **Total** | 60 | 11 | 47 | 27 | 144 | 144 |
|  | 289 | | | | |  |

^*^ Median ± SD.

Patients suffered from falciparum malaria (FM); healthy individuals (Healthy)
